# GSK-3 inhibitors enhance TRAIL-mediated apoptosis in human gastric adenocarcinoma cells

**DOI:** 10.1371/journal.pone.0208094

**Published:** 2018-12-17

**Authors:** Yi-Ying Wu, Chin-Tung Hsieh, Ying-Ming Chiu, Shen-Chieh Chou, Jung-Ta Kao, Dong-Chen Shieh, Yi-Ju Lee

**Affiliations:** 1 Department of Medical Laboratory Science and Biotechnology, China Medical University, Taichung, Taiwan; 2 Chinese Medicine Research Center, China Medical University, Taichung, Taiwan; 3 Research Center for Chinese Herbal Medicine, China Medical University, Taichung, Taiwan; 4 Department of Pediatrics, Lo-Hsu Medical Foundation Lotung Poh-Ai Hospital, I-Lan, Taiwan; 5 Department of Nursing, College of Medicine and Nursing, Hungkuang University, Taichung, Taiwan; 6 Division of Allergy, Immunology & Rheumatology, Changhua Christian Hospital, Changhua, Taiwan; 7 Department of Biological Science and Technology, School of Medicine, China Medical University, Taichung, Taiwan; 8 Graduate Institute of Clinical Medical Science, China Medical University, Taichung, Taiwan; 9 Department of Internal Medicine, School of Medicine, China Medical University Hospital and China Medical University, Taichung, Taiwan; 10 Institute of Biochemistry, Microbiology and Immunology, Chung Shan Medical University, Taichung, Taiwan; Institute of Biochemistry and Biotechnology, TAIWAN

## Abstract

Resistance to tumor necrosis factor-related apoptosis-inducing ligand (TRAIL)-induced apoptosis has been reported in some cancer cells, including AGS human gastric adenocarcinoma cells. Reducing this resistance might shed light on the treatment of human gastric adenocarcinoma. In this study, we examined whether glycogen synthase kinase-3 (GSK-3) inhibitors can restore TRAIL responsiveness in gastric adenocarcinoma cells. The effect of two GSK-3 inhibitors, SB-415286, and LiCl, on apoptosis signaling of TRAIL in human gastric adenocarcinoma cell lines and primary gastric epithelial cells was analyzed. Both inhibitors can sensitize gastric adenocarcinoma cells, but not primary gastric epithelial cells, to TRAIL-induced apoptosis by increasing caspase-8 activity and its downstream signal transmission. Adding p53 siRNA can downregulate GSK-3 inhibitor-related sensitization to TRAIL-induced apoptosis and caspase-3 activity. GSK-3 inhibitors strongly activate the phosphorylation of JNK. Inhibition of JNK leads to earlier and more intense apoptosis, showing that the activation of JNK may provide anti-apoptotic equilibrium of pro-apoptotic cells. Our observations indicate that GSK-3 inhibitors can sentize AGS gastric adenocarcinoma cells to TRAIL-induced apoptosis. Therefore, in certain types of gastric adenocarcinoma, GSK-3 inhibitor might enhance the antitumor activity of TRAIL and mightbe a promising candidate for the treatment of certain types of gastric adenocarcinoma.

## Introduction

Gastric cancer is a malignant neoplasm with poor prognosis [1]. Surgical treatment is currently the only option which offers curative potential to patients with locally advanced gastric cancer [2]. Tumor necrosis factor-related apoptosis-inducing ligand (TRAIL), a novel TNF superfamily member with strong homology to FasL, is capable of inducing apoptosis in a variety of transformed cell lines *in vitro* [3]. Recent studies indicated that TRAIL-induced apoptosis occurred through a caspase signaling cascade [4]. Resistance to TRAIL-induced apoptosis has been reported in some cancer cells, including AGS human gastric adenocarcinoma cells [5–6]. Therefore, the restoration of TRAIL-induced apoptosis sensitivity might offer a new treatment option for human gastric adenocarcinoma.

Lately, the glycogen synthase kinase-3 (GSK-3) has been defined as a negative regulator of TNF-signaling. Recently, GSK-3 inhibitors have been found to promote TRAIL- and CD95-induced apoptosis in colon cancer cells, Jurkat and pancreatic cancer cells [7].

Interestingly, it is unclear whether GSK-3 inhibitors can influence the sensitivity of untransformed and transformed gastric epithelial cells to TRAIL- and CD95L-induced apoptosis. The purpose of this study was to identify whether the GSK-3 inhibitors restore the sensitivity of cancer cells to CD95- and TRAIL-induced apoptosis.

## Materials and methods

### Cell culture

Human gastric adenocarcinoma cell line AGS was obtained from ATCC and maintained in DMEM, supplemented with 10% FBS [8–11]. Human primary gastric epithelial cells were cultured using our previously described method [11–14]. The study was approved by the Institutional Review Board of the China Medical University Hospital (Taichung, Taiwan) and assigned the protocol number of DMR97-IRB-263.

### Flow cytometry analysis

Apoptotic cells stained were detected in the sub-G1 peak as our previously described method [13–14]. Both adherent and floating cells were collected, washed, fixed in 70% ethanol at -20°C and stained with 20 μg/mL propidium iodide (Sigma–Aldrich) in the presence of 100 μg/mL ribonuclease A (Sigma–Aldrich) for 30 min at 37°C in the dark. DNA content was analyzed by flow cytometry (FACS Calibur, Becton Dickinson, Mountain View, CA). Apoptotic cells with hypodiploid DNA staining were detected in the sub G1 peak.

### Western blotting

Whole-cell lysates were prepared as we previously described [11, 15]. Proteins were resolved on SDS-PAGE gels and were then transferred to Immobilon polyvinyldifluoride (PVDF) membranes. The blots were blocked with 5% non-fat dry milk in Tris-buffered saline containing 0.5% Tween-20 (TBST) for 1 h at room temperature and were then probed with rabbit anti-mouse antibodies for 1 h at room temperature. After three washes, the blots were incubated with a donkey anti-rabbit peroxidase-conjugated secondary antibody (1:1000) for 1 h at room temperature. The blots were visualized with enhanced chemiluminescence using Kodak X-OMAT LS film (Eastman Kodak) [16].

### Reagents

TOOLStripping Buffer, TW-ST500 (TOOLS, Taiwan), SB-415286 (Bender MedSystems, Burlingame, CA), Camptothecin, LiCl (Pierce Biotechnology, Rockford, IL), recombinant human TRAIL (Tocris, Ellisville, MO). Clone CH-11 (anti-CD95 Antibody, Upstate, Charlottesville, VA), Common Caspase Inhibitor Z-VAD-fmk(Calbiochem, Darmstadt, Germany), Caspase-8 Inhibitor ZIETD-fmk, SP600125 (San Diego, CA), AS601245 (R&D Systems, Minneapolis, MN). Nuclear and cytoplasmic extracts were prepared by using the NE-PERTM kit (Sigma-Aldrich, St Louis, MO). Phosphorylated Ser9-GSK-3', pan Akt phospho Ser473, total AKT monoclonal antibody, phosphorylated Ser641-GS, cleaved caspase-8, -9 and -3 bid, phosphorylated Thr183 / Tyr185-JNK, JNK, P53 (BD Biosciences PharMingen), GSK-3β(Imgenex, San Diego, CA), TRAIL-R1 (Cell Signaling Technology, Danvers, MA), TRAIL-R2 (Upstate Biotechnology), bax and ADP ribose polymerization PARP(BIOMOL Research Laboratories, Plymouth Meeting, PA). Caspase-3 activity was calculated by cleaving a colorimetric substrate (Ac-DEVD-pNA) (Santa Cruz, Biotechnology). The p53 siRNA was synthesized by Ambion (Austin, TX) against the following sequence 5'-GCAUGAACCG-GAGGCCCAUTT-3' in p53 mRNA. Unrelated siRNA sequences were used as controls (Ambion). SiRNA-GSK-3 (Santacruz biotechnology, Dallas, TX).

### Using siRNA strategy to downregulate p53/ GSK-3 expression

AGS incubated in 12-well plates (3 x 10^5^ cells/well) were grown for 24 hours to 50% confluence according to the manufacturer’s instructions and then transfected with 75 nM siRNA using LipofectAMINE 2000 (InVitrogen Corp.). After 24 hours, cells were washed with PBS and restimulated with or without TRAIL and/or LiCl for 24 hours prior to collection for protein extraction or caspase-3 activity test as previously reported [17].

### Synergism experiments

Drug-drug interactions between SB-415286 or LiCl and TRAIL were evaluated in AGS cells by MTT assay. Cells were incubated with each drug independently and in combination for 72 h before assessment of cytotoxicity. The combination index (CI) was produced by Compusyn software (version 1.0.1), where CI < 1 indicate synergism, CI = 1 designate additive effect, and CI > 1 means antagonism [18]. Cells were then cultured in 75-cm^2^ flasks to adhere for 24 h under routine conditions. After this period, each three of 75-cm^2^ flasks containing cells were incubated with SB-415286 or LiCl and TRAIL for 72 h.

### Statistical analysis

All data were analyzed using SPSS 15.0 for Windows [13, 19]. P<0.01 was considered statistically significant in all comparisons. Blots were quantified using PRISM 4.0 (GraphPad Software Inc, San Diego, CA, USA) [11, 13, 15, 20, 21].

## Results

### Effects of glycogen synthase kinase-3 inhibitor on apoptosis induced by CH-11 and tumor necrosis factor-related apoptosis-inducing ligand (TRAIL) in gastric adenocarcinoma cell Line AGS

**Fig 1:**
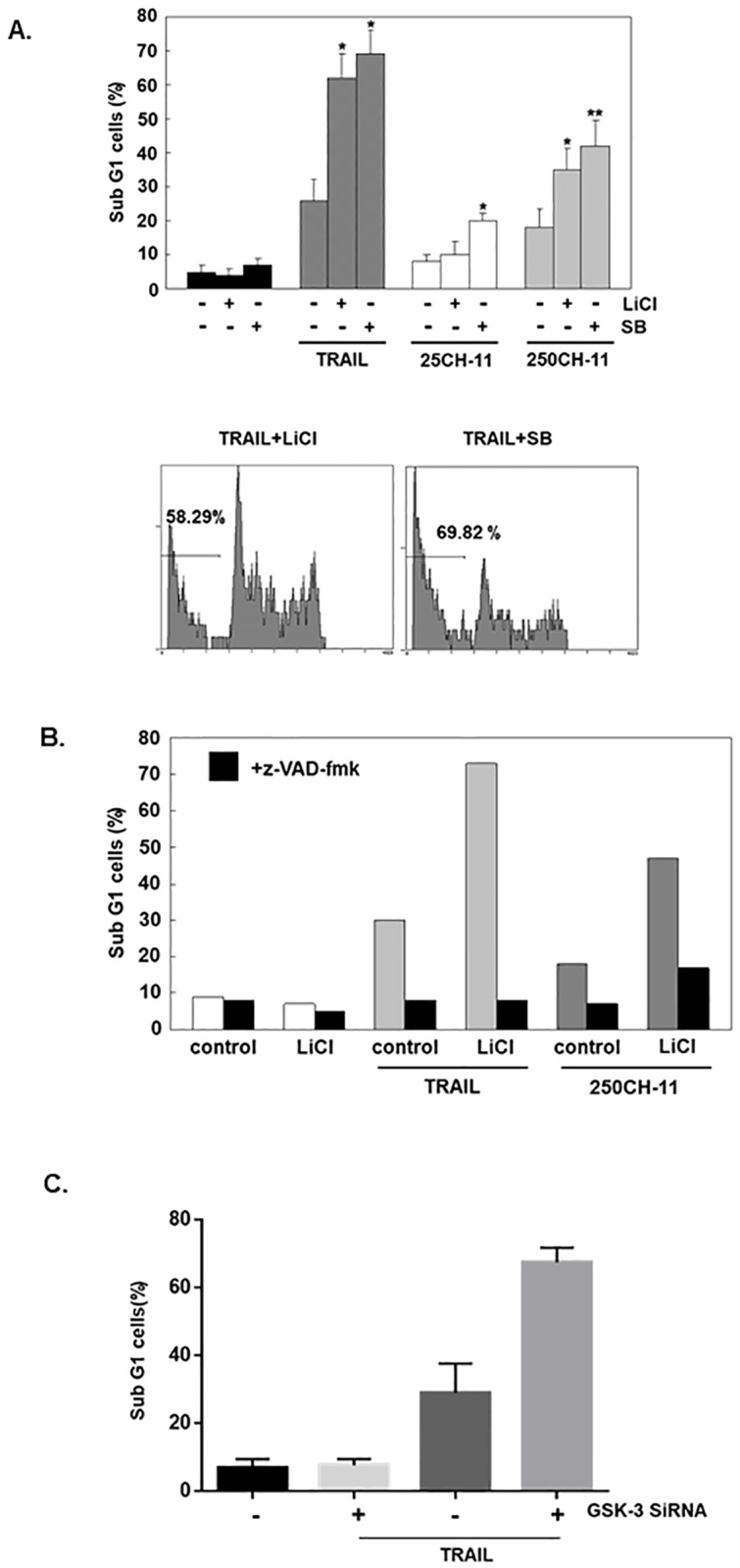
Effects of GSK-3 inhibitor on apoptosis induced by agonistic anti-CD95 and TRAIL in gastric adenocarcinoma cell line AGS. (A) AGS cells were treated with or without TRAIL (25 ng/mL), CH-11 (agonistic anti-CD95 antibodies) (25 ng/mL or 250 ng/mL), LiCl (20 mM) or SB (25 μM). Flow cytometry analysis was used to analyze sub-G1 cell populations. The results are shown as mean ± the standard error (SE) of four independent experiments. (B) AGS cells were incubated with TRAIL (25 ng/mL), agonistic anti-CD95 antibodies (250 ng/mL), LiCl (20 mM) or z-VAD-fmk for 24 hours (20 μM). Then sub-G1 cells were analyzed using flow cytometry. The result was the mean of three independent experiments. The statistical significance was determined by comparison with cells treated with DR (death receptor) agonist only (*, p <0.05; **, p < 0.01). (C) siRNA against GSK3 increases the TRAIL cytolytic killing of AGS cells (n = 5).

The outcomes of two widely used glycogen synthase kinase-3 inhibitors, SB-415286 and LiCl, on TRAIL or CD95 agonistic antibody (CH-11)-induced AGS cell apoptosis were examined, and sub G1 population were analyzed by flow cytometry. SB-415286 and LiCl are used with the concentrations of 25 μM and 20 mM, respectively (higher than the IC50 [22, 23] to obtain effective intracellular concentrations in intact cells), for in vitro experiments. After 24 hours, the combination of SB-415286 or LiCl with 25 ng/mL TRAIL (low-dose) significantly enhanced the percentage of sub-G1 population (from 4.8 ± 0.6% to 60.7 ± 7.2% and 68.6 ± 9%, respectively), while TRAIL alone can enhance the percentage to 27.8±6% (Fig 1A). For low concentrations of CD95 agonistic antibody (25ng/mL), only CD95 agonistic antibody together with SB-415286 increased the percentage of sub-Gl population (20.6±2.2%) (Fig 1A). Interestingly, with high dose (250 ng/mL) of CD95 agonistic antibody, the sub-G1 cell ratios of adding lithium chloride or SB-415286 were 32.3±6.4% and 45.7±7.5%, respectively, while CD95 agonistic antibody alone enhenced cell death to 19± 5.4% (Fig 1A). These results suggest that GSK-3 inhibitors can sensitize the AGS cells to the cytotoxic effects of CD95 agonistic antibody and TRAIL. The z-VAD-fmk (pan-caspase inhibitor) prevented lithium sensitization of TRAIL- and CD95 agonistic antibody-induced apoptosis (Fig 1B), demonstrating that both reactions involve apoptotic mechanisms.

We then repeated the same experiment by using GSK-3 siRNA to knock down its expression to avoid the off-target effects by the inhibitors and similar result was obtained (Fig 1C).

### Outcomes of glycogen synthase kinase-3 inhibitor in apoptosis induced by CH-11 and TRAILin primary human gastric epithelial cells

**Fig 2:**
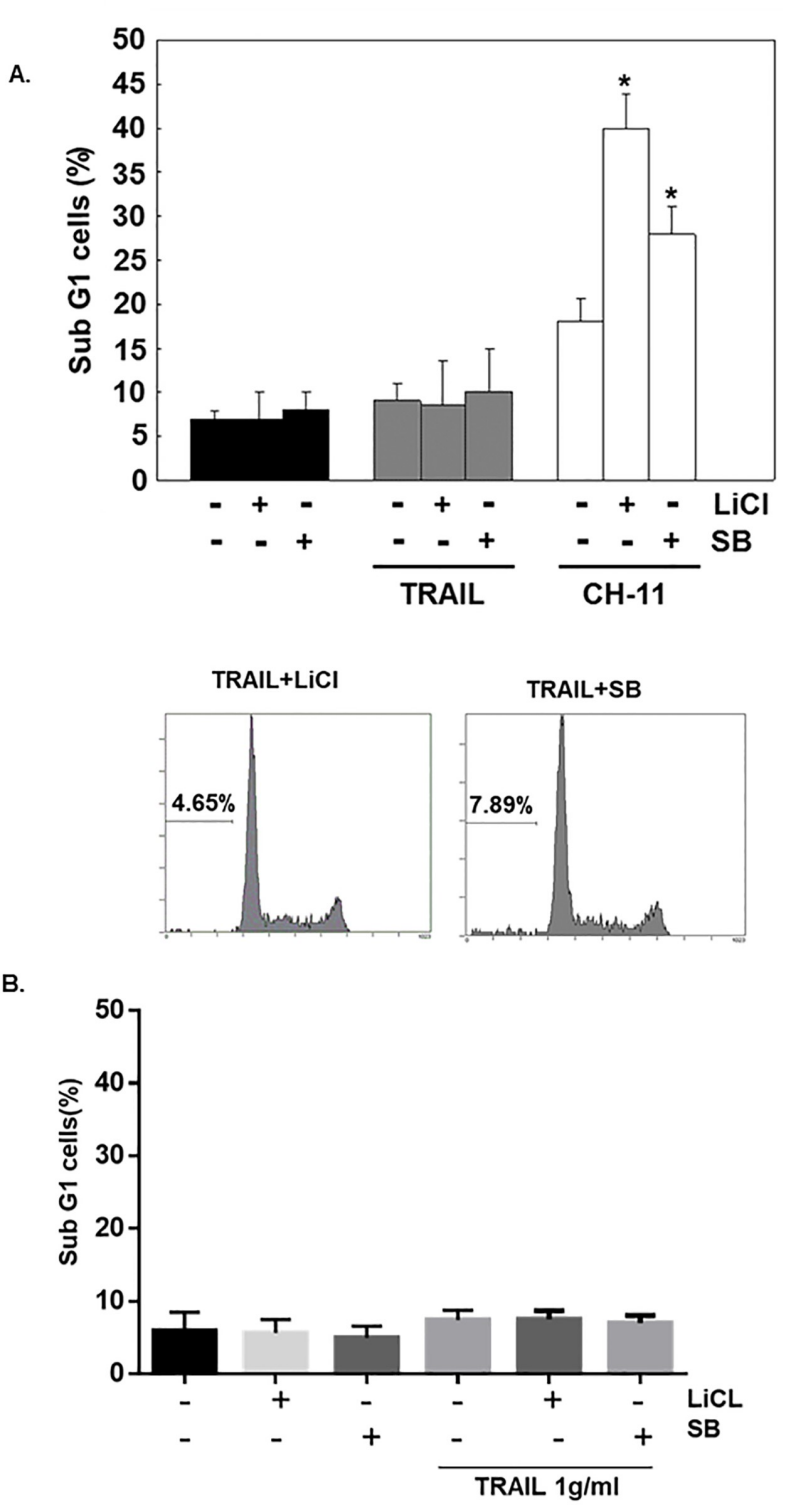
Effects of GSK-3 inhibitor on apoptosis induced by agonistic anti-CD95 and TRAIL in primary human gastric epithelial cells. (A) Treatment of normal human primary gastric epithelial cells with or without TRAIL (25ng/mL), agonistic anti-CD95 antibodies (25ng/mL), LiCl (20mM) or SB (25μM) for 24 hours. Then sub-G1 cells were analyzed by flow cytometry. The result was the mean of three independent experiments. The statistical significance was determined by comparison with primary gastric epithelial cells incubated with agonistic anti-CD95 antibodies only (*, p < 0.01). (B) Treatment of normal human primary gastric epithelial cells with or without TRAIL (1mg/mL), LiCl (20mM) or SB (25μM) for 24 hours. Then sub-G1 cells were analyzed by flow cytometry. The result was the mean of three independent experiments.

Next we examined whether similar phenomenon would be observed in human primary gastric epithelial cells. In primary human gastric epithelial cells, CD95 agonistic antibody enhanced apoptosis (from 7.2±0.8% to 18.0±2.6%), whereas TRAIL (25ng/mL) was ineffective (Fig 2A). Lithium chloride or SB-415286 alone is non-toxic, but they significantly increase CD95 agonistic antibody -induced apoptosis (2.2 and 1.6 fold, separately), and they could not overcome the resistance of primary human gastric epithelial cells to TRAIL-induced apoptosis. We increased the dose of TRAIL to 1.0 g/mL but still could not induced apoptosis in primary human gastric epithelial cells (Fig 2B) [6]. This phenomenon indicates primary human gastric epithelial cells are not responsive to TRAIL at all.

### Phosphorylation of AKT and glycogen synthase kinase-3 are not modified by GSK-3 inhibitorduring TRAIL-induced apoptosis sensitization

**Fig 3:**
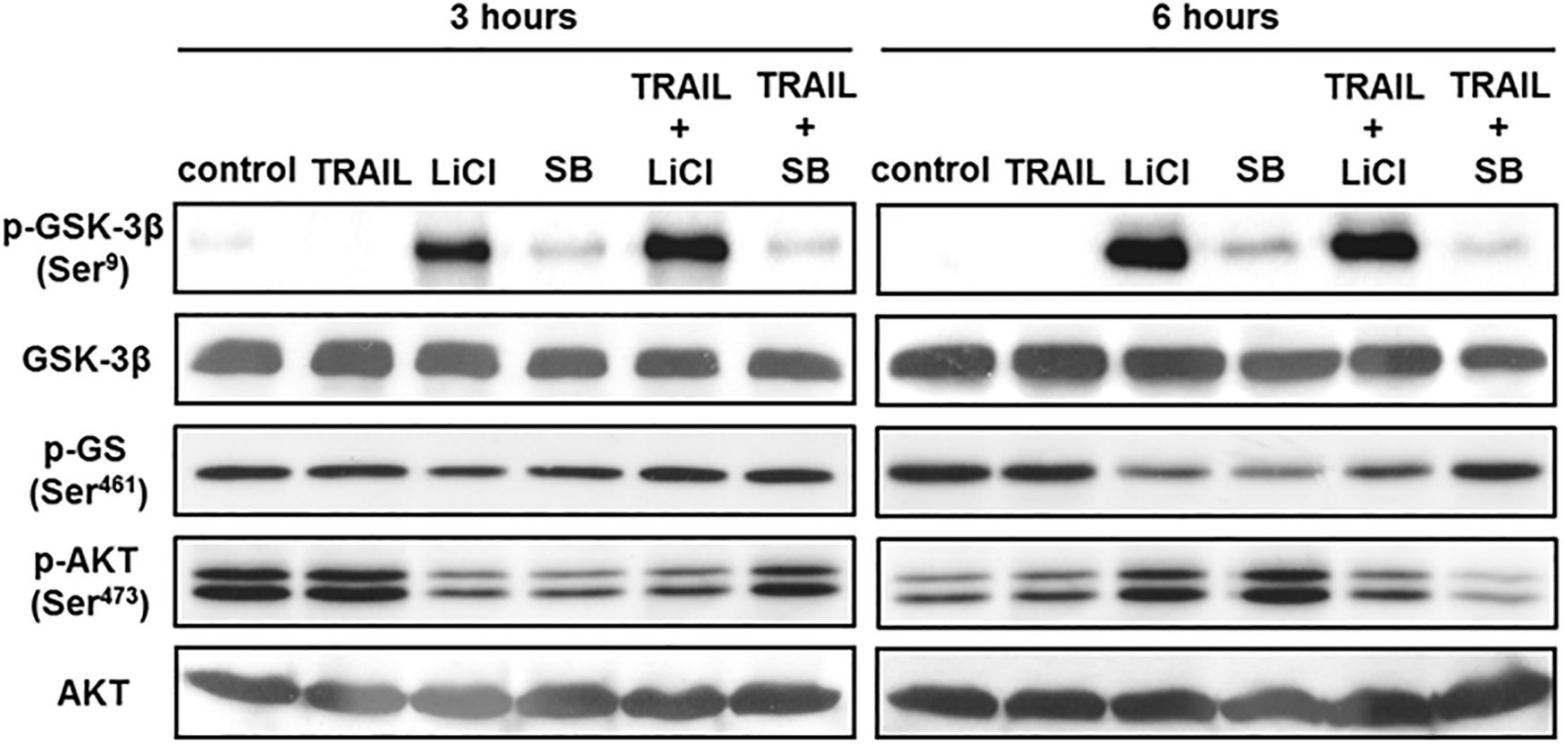
Phosphorylation of GSK-3 and AKT are not modified by GSK-3 inhibitor during its sensitization of TRAIL-induced apoptosis. AGS cells were treated with or without TRAIL (25 ng/mL), LiCl (20 mM) or SB (25 μM) for 3 hours and 6 hours. GS, AKT and GSK-3β phosphorylation expression were analyzed by Western blotting of AGS whole extracts. AKT was detected by total AKT monoclonal antibody, which detects endogenous levels of AKT and does not cross-react with related proteins. We used pan Akt phospho Ser473 to enable the homogeneous detection of all phospho Akt isoforms for oncology. Blots are representative of four independent experiments.

To measure GSK-3 activity during combined treatment of TRAIL and SB-415286 or LiCl, we observed the phosphorylation of the inhibitory serine 9 residue of the GSK-3β isoform, which parallel with the level of inactivation of the enzyme, Glycogen synthase (GS), the direct substrate, and phosphorylation level of GSK-3. AGS were treated with SB-415286, LiCl or TRAIL for 3 and 6 hours and then whole cell extracts were obtained for analysis. Lithium chloride and SB-415286 inhibit GSK-3 through different mechanisms. LiCl was a Mg^2+^ rival inhibitor and enhence serine 9 phosphorylation of glycogen synthase kinase-3 [24]. SB-415286 acts as an ATP rival inhibitor without affecting GSK-3 phosphorylation [25]. Therefore, we noticed that LiCl, but not SB-415286, enhenced the phosphorylation of GSK-3 Ser9 in AGS cells (Fig 3). All GSK-3 inhibitors reduced glycogen synthase phosphorylation, showing that glycogen synthase kinase-3 is significantly suppressed under these conditions. After incubated with TRAIL, there was no further change in the phosphorylation status of GSK-3 and GS. We also checked the status of total AKT by total AKT monoclonal antibody, which detects endogenous levels of AKT and does not cross-react with related proteins. We used pan Akt phospho Ser473 to enable the homogeneous detection of all phospho Akt isoforms for oncology. No alteration of AKT phosphorylation and expression was found under any of the conditions tested (Fig 3).

### Sensitization effect of glycogen synthase kinase-3 inhibitor needs enhancement of Caspase-8 activity in AGS

**Fig 4:**
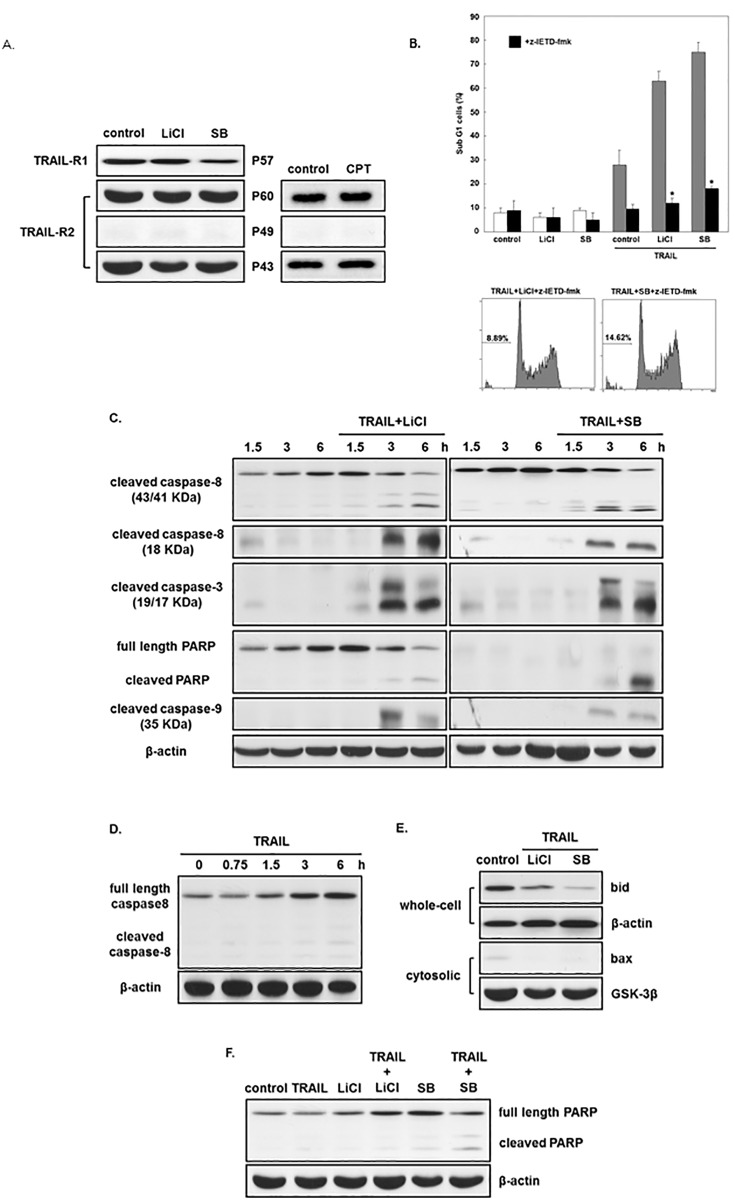
Sensitization effect of GSK-3 inhibitors requires enhancement of caspase-8 activity. (A) AGS cells were incubated with or without LiCl (20 mM) or SB (25 μM) for 24 hours. Western blot analysis of TRAIL-Rl and TRAIL-R2 expression of whole cell extracts (40 μg) was analyzed. AGS incubated with CPT (0.5μ M) for 12 hours served as a positive control (camptothecin) for upregulation of TRAIL-R2. (B) Blots are representative of four independent experiments and the quantified values are the mean ±the standard error (SE) of four independent experiments. The statistical significance was determined by comparing with AGS treated with LiCl/TRAIL or TRAIL/SB-415286, *, p < 0.01. AGS incubated with or without TRAIL (25 ng/mL), LiCl (20 mM), SB (25 μM) and or z-IETD-fmk (caspase-8 inhibitor, 20 μM) for 24 hours. Then Sub-G1 cells were analyzed by flow cytometry. (C and D) AGS were incubated with or without TRAIL (25 ng/mL), LiCl (20 mM) or SB (25 μM) for 0.75, 1.5, 3 and 6 hours. Caspase-8, -3, -9 and PARP-cleaved in AGS whole extracts were evaluated by Western blotting. (E) AGS were incubated for 6 hours with or without TRAIL (25 ng/mL), LiCl (20 mM) or SB (25 μM). The AGS whole extracts were analyzedfor bid expression and evaluated for bax expression in cytosolic extracts by Western blotting. Replication was performed with GSK-3β and β-actin Abs to confirm equivalent loading. (F) AGS were treated with or without TRAIL (25 ng/mL), LiCl (20 mM) or SB (25 μM) for 6 hours. AGS whole extracts were evaluated for PARP cleavage by Western blotting.

We next investigated the mechanism underlying the effect of GSK-3 inhibitors in TRAIL-induced cell apoptosis in AGS. We examined the effect of GSK-3 inhibitors on TRAIL-R1 and TRAIL-R2 levels. Three isoforms of TRAIL -R2 were examined in AGS: the p43, p49 and p60 form (Fig 4A). TRAIL-R1 and TRAIL-R2 levels did not increase after incubation with SB-415286 or LiCl (Fig 4A) for 24 hours, showing that GSK-3 inhibitor-induced sensitization of TRAIL-induced apoptosis in AGS is not related to upregulation of TRAIL receptors.

Next, we studiedthe signaling pathway induced by TRAIL and investigated if the sensitization of GSK-3 inhibitors involves activation of caspase-8. Pretreatment with the z-IETD-fmk (caspase-8 inhibitor) diminished the cell death inducedby TRAIL with SB-415286 or LiCl (Fig 4B), showing that GSK-3 inhibitor activates caspase-8. AGS was incubated with TRAIL and SB-415286 or LiCl for 3 hours(Fig 4C). We demostrated a significant increase of caspase-8 cleavage while treated with TRAIL (Fig 4D). The combination treatment of TRAIL and GSK-3 inhibitor also increased the cleavage of PARP and caspase-3 (Fig 4C). GSK-3 inhibitor sensitization involves a mitochondrial pathway because the combined treatment promotes free lysis and the disappearance of bax from the cytosol through caspase-9 activation (Fig 4C and 4E). The combination treatment did not induce PARP cleavage in AGS (Fig 4F). Taken together, we demonstrated that GSK-3 inhibitors can sensitize AGS cells to TRAIL-mediated apoptosis. GSK-3 inhibits TRAIL-induced apoptosis might be one of the possible mechanism underline this phenomenon. However, more experiments are still needed to elucidate the exact mechanism in the future.

### P53 participates in the sensitization of GSK-3 inhibitors on TRAIL-induced apoptosis

We next examined the role of GSK-3 inhibitors to the expressionof p53 in TRAIL-induced apoptosis, as GSK-3 modulates p53 enrichment and its effect [26].

**Fig 5:**
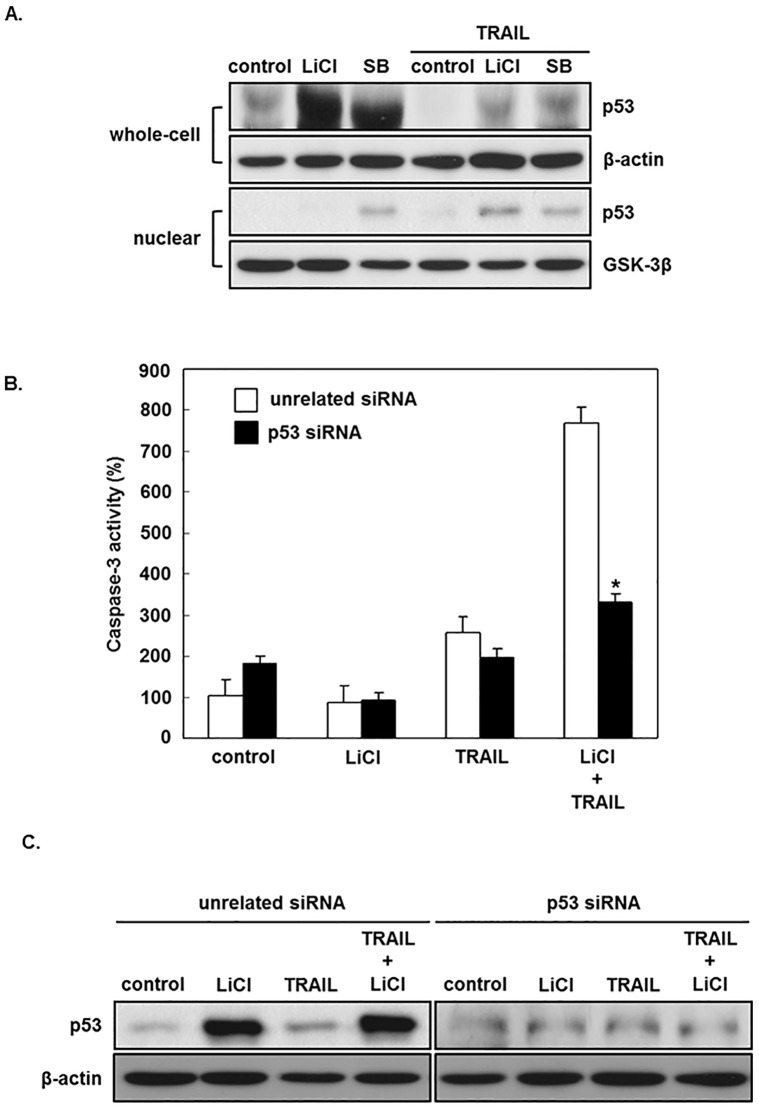
P53 participates in the sensitization effect of GSK-3 inhibitors on TRAIL-induced apoptosis. (A) AGS were incubated with or without TRAIL (25 ng/mL), SB-415286 (25 μM) and/or LiCl (20 mM) for 12 hours. Nuclear extracts and whole cell were analyzedfor p53 expression by Western blot. AGS were transfected with p53 or irrelevant siRNA and treated with or without TRAIL (25 ng/mL) and LiCl (20 mM) for 24 hours and then evaluated for p53 expression by Western blot (B) and colorimetric determination of caspase-3 activity (C). Representative blots of four independent experiments are shown. The results are shown as the mean ±the standard error (SE) of four independent experiments. *, p < 0.05 when compared to cells transfected with irrelevant siRNA.

TRAIL alone had no influence on p53 expression (Fig 5A). In Fig 5A, the Western blotting of p53 in nuclear component was increased after TRAIL and LiCl treatment compared with LiCl treatment alone. However, Western blotting of p53 in nuclear component was increased after SB treatment, with or without TRAIL. Our results indicate that the effect of SB on p53 is independent of the presence or absence of TRAIL.

We next examined the effect of p53 siRNA on AGS cells treated with LiCL and TRAIL(Fig 5B and 5C). For sensitive measurement of apoptosis, a colorimetric assay was used to assess caspase-3 activity. Transient transfection of p53 siRNA resulted in remarkable down-modulation of p53 in the AGS cells either treated with LiCl only or treated with LiCl and TRAIL(Fig 5C). While p53 siRNA has no influence on caspase-3 activity when treated with TRAIL alone, it remarkably reduces caspase-3 activation enhanced by treating with TRAIL together with LiCl (Fig 5B), showing that p53 is involved in LiCl-induced sensitization of TRAIL–induced apoptosis.

### JNKs limit the extent of GSK-3 inhibitor-induced sensitization to TRAIL -induced cell death

**Fig 6:**
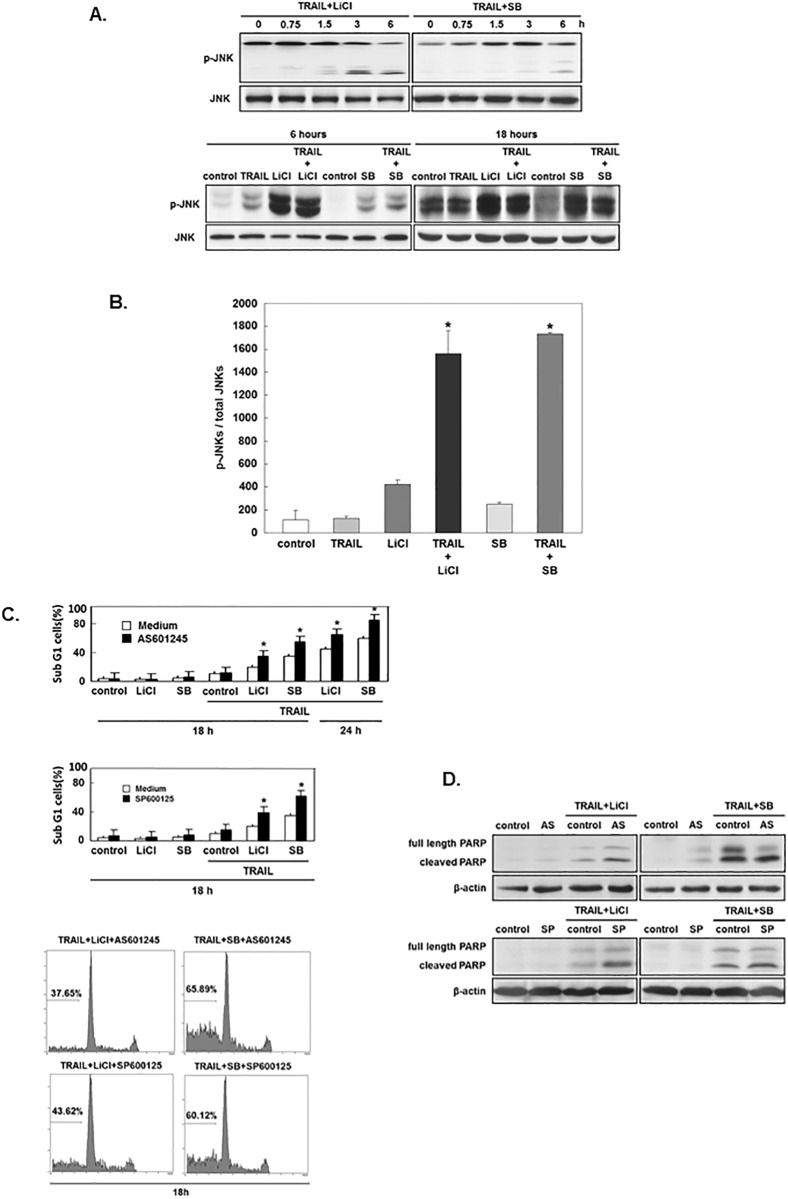
JNKs limit the extent of GSK-3 inhibitor sensitization of TRAIL-induced apoptosis. (A) AGS cells were treated with or without TRAIL (25 ng/mL), lithium (LiCl, 20 mM) and/or SB-415286 (SB, 25 μM) for 18 hours. Total AGS extracts (40 μg) were analyzed for phosphorylation and total level of JNK by Western blotting. (B) 6 hours of treatment and calculation of phosphorylated form/total JNK ratio. The quantitative value is the mean ± the standard error (SE) of four independent experiments. *, p < 0.05 compared to cells treated with GSK-3 inhibitor only. (C) LiCl (20 mM), SB (25 μM) or JNK inhibitor (AS601245 2 μM or SP600125 5 μM), with or without TRAIL (25 ng/mL) of sub-G1 cells. Blots are representative of three independent experiments and the results are referenced to ±the standard error (SE). *, p < 0.05 compared to cells treated with GSK-3 inhibitors and TRAIL in four independent experiments. (D) AGS cells treated with or without LiCl (20 mM), SB (25 μM), JNK inhibitor (AS601245 2 μM or SP600125 5 μM), or TRAIL (25 ng/mL). PARP cleavage was analyzed by Western blotting.

The combination of TRAIL with SB-415286 or LiCl significantly enhanced c-Jun N-terminal kinase phosphorylation without altering c-Jun N-terminal kinase expression (Fig 6A). C-Jun N-terminal kinase activation was examined at 0.75 hours (early time point) and maintained for at least 18 hours (Fig 6A). TRAIL alone can not activate JNK, while Lithium or SB-415286 alone can increase JNK activity. However, JNK activation was greatly enhanced when treated with TRAIL and GSK-3 inhitors (Fig 6A and 6B). To test the role of JNK in GSK-3 inhibitor-induced sensitization of TRAIL-induced apoptosis, we added JNK inhibitor AS601245 with TRAIL and SB-415286 or LiCl for 24 hours and the level of apoptosis was significantly enhanced when compared with AGS treated with SB 415286/TRAIL or LiCl /TRAIL [27] (Fig 6C). At 18 hours (earlier time point), adding SP600125 or AS601245 [28] resulted in notable and strong enhancement of cell death. c-Jun N-terminal kinase inhibitor alone had no activity on cell death and did not alter the cellular responseto SB-415286 or LiCl. Assessment of PARP cleavage after 6 hours of treatment demonstrated that the JNK inhibitor enhanced apoptosis signaling induced by LiCl /TRAILor SB-415286/TRAIL (Fig 6D). Our observation suggests GSK-3 inhibitors activate JNK, which in term limits the extent of cell death enhanced by TRAIL/ LiCl or TRAIL/SB-415286.

## Discussion

In our study, we demonstrate that GSK3 inhibitors could sensitize certain gastric adenocarcinoma cells to TRAIL-induced apoptosis by increasing caspase-8 activity and its downstream signal transmission.

CompuSyn software was used to determine the type of drug interaction between TRAIL and GSK-3 inhibitors. Table 1 presents the combination indices (CIs) detected after treatment of AGS cells with different combinations of the two agents and indicated their interaction pattern. The CI values were estimated according to the method of using CompuSyn software, where CI < 1, CI = 1, and CI > 1 designated synergism, additive effect, and antagonism, respectively [18]. The results in Table 1 showed the addition of 25 μM SB-415286 to TRAIL has a CI of 0.92, which showes synergistic effect. Treatment with LiCl 20mM and TRAIL has a CI of 0.89, which also favors synergism. (Table 1).

**Table 1:** Combination index (CI) values of adding SB-415286/ LiCl to TRAIL in combination in AGS cells.

TRAIL	GSK-3 inhibitors	combination index (CI)
25 ng/mL	LiCl(25mM)	0.89
25 ng/mL	SB-415286(20μM)	0.92

AGS cells were treated with the combination of SB-415286 or LiCl to TRAIL at doses indicated. CompuSyn software was used to analyze the data and calculate the CI value, where CI<1, CI = 1, and CI>1 indicated synergism, additive effect, and antagonism, respectively. The CI = (dA/DA)+(dB/DB), where dA and dB are the concentrations of SB-415286/ LiCl and TRAIL in combination, whereas, DA and DB are the concentrationsof SB-415286/ LiCl or TRAIL, respectively, which produce the same effect alone.

TRAIL has become a promising anticancer drug because it selectively increases cell death in a wide range of cancer cells without toxicity to normal cells [29]. TRAILis not toxic to primary gastric epithelial cells [6] as showed in our previous study and the current study. We show that there is a compensatory anti-apoptotic signaling pathway in gastric adenocarcinoma cells, which might plays a role in the resistance to TRAIL-induced apoptosis.

We illustrate that GSK-3 inhibitors significantly increase TRAIL-induced cell death in gastric adenocarcinoma cells without affecting primary gastric epithelial cells. GSK-3 inhibitors sensitize not only TRAIL-induced, but also CH-11-induced apoptosis, making itself a potential candidate of cancer treatment in addition to its previously reported role in anti-inflammation [30].

Upregulation of TRAIL receptors has been reported as a mechanism to increased TRAIL cytotoxicity in some studies [31] but not all [32]. In our study, Caspase-8 inhibitors can eliminate GSK-3 inhibitors-induced sensitization to TRAIL-induced caspase-8 activation and apoptosis, which was compatible to previous reports [33].

The strong pro-apoptotic effect of GSK-3 inhibitors in extrinsic pathways triggered by death receptors has been well documented in our study and previous studies in other cancer cell types [34]. Selective knockdown of glycogen synthase kinase-3β but not glycogen synthase kinase-3α by RNA interference enhances TRAIL-induced cell death in pancreatic cancer cells [35]. We demonstrated that this process might implicate tumour suppressor p53. Although NF-kB might be involved in the sensitization to TRAIL signaling induced by GSK-3 inhibitors, it has been excluded by others [36].

We have shown that GSK-3 inhibitors activate JNK in gastric adenocarcinoma cells, which regulate negatively on TRAIL-induced apoptosis, thereby providing a anti-apoptotic signal. These findings also suggest that GSK-3 might have a negative effect on JNK activation, which is consistent with a previous study about lithium chloride-induced JNK activation in neurons [37] and observations noted in glycogen synthase kinase-3 knockout embryonic fibroblasts [38]. In gastric adenocarcinoma cells, since JNK activation occurs at an earlier time point, it is not related to caspase activation. In summary, our results support the concept that glycogen synthase kinase-3 sets the threshold for apoptosis induction by modulating the concomitant proapoptotic and antiapoptotic signals [39].

Besides cancers, GSK-3 inhibitors are currently under studies for its potential clinical usages in many fields [40]. In summary, we show that GSK-3 inhibitors sensitize gastric adenocarcinoma cells but not primary gastric epithelial cells to TRAIL-induced apoptosis by inducing caspase-8 activation through mitochondrial pathway. This cancer cell-specific nature might be beneficial for the clinical application of combined GSK-3 inhibitor and TRAIL therapy for certain types of gastric cancer patients in the future.

## Abbreviations

CH-11: agonistic anti-CD95 antibodies
DR: death receptor
GSK-3: glycogen synthase kinase-3
JNK: c-Jun N-terminal kinase
JNKs: c-Jun N-terminal kinases
LiCl: lithium chloride
SB: SB-415286
TNF-alpha: tumor necrosis factor-alpha
TRAIL: tumor necrosis factor-related apoptosis-inducing ligand
z-IETD-fmk: caspase-8 inhibitor
z-VAD-fmk: pan-caspase inhibitor
